# The Evolution and expression analysis of USP gene family in *Solanum*

**DOI:** 10.3389/fpls.2025.1546640

**Published:** 2025-06-30

**Authors:** Ruiqiang Xu, Zhongyu Wang, Zhaolong Chen, Zepeng Wang, Qingyuan Meng, Ning Li, Yong Qin

**Affiliations:** ^1^ College of Horticulture, Xinjiang Agricultural University, Urumqi, Xinjiang, China; ^2^ Biological Breeding Laboratory, Xinjiang Uygur Autonomous Region Academy of Agricultural Sciences, Urumqi, China

**Keywords:** USP gene family, tomato, domestication, structural variants, expression

## Abstract

As natural environments degrade and extreme weather events become more frequent, humanity increasingly faces the challenge of producing crops under various complex and adverse conditions. Improving crop adaptability has become crucial. Universal stress proteins (USPs) are a class of small molecular proteins widely found in plants, capable of withstanding various biotic and abiotic environmental stresses, including temperature stress, drought, nutrient deficiency, oxidative imbalance, salt and heavy ion toxicity, and pathogenic infections. Enhancing our understanding of USPs holds significant potential for improving plant stress resilience. This study focuses on 13 species of *Solanum*, including cultivated and wild tomatoes, and systematically identified 438 members of the USP gene family through bioinformatics approaches. Phylogenetic analysis reveals that major USP members are conserved within *Solanum*, with interspecies differences in USP numbers primarily attributed to copy number variation (CNV). Through synteny and homology analyses, we found that *USP27* and *USP28* are unique to tomatoes, while the homologous gene of *USP19* is absent in cultivated tomatoes. Notably, five unique USP genes are present in *S. pennellii*, which is characterized by its early differentiation and resistance advantages. *Ka*/*Ks* analysis indicates that only the *USP10*/*21* homologous gene pair has undergone positive selection in wild tomatoes, while all other genes are subject to strong negative selection. The USPs in *Solanum* exhibit high consistency in domain characteristics, sequence conservation, and types of promoter regulatory elements, although there are substantial differences in the number of these elements. Utilizing publicly available data, we identified eight USPs that have undergone domestication or improvement selection, particularly noting the tissue-specific expression patterns of domesticated *SolycUSP3/28/30*. Through graph pangenome analysis, we screened 12 *USPs* covered by high-confidence structural variants, which primarily disrupt the intron regions of *USPs*, leading to significant differences in their expression responses to salt stress. We anticipate that these findings will provide a theoretical foundation and prior knowledge for further understanding and application of USP in plants.

## Introduction

The rapid changes in the global environment and the increasing frequency of extreme weather events pose significant challenges to plant growth and crop production. Understanding the stress and defense systems that plants have evolved to cope with mixed and complex adverse environmental conditions is crucial for enhancing and improving plant adaptability. Universal stress proteins (USPs) are small molecular proteins that exhibit responses and resistance to multiple stressors; they were initially identified in *Escherichia coli* (Vanbogelen et al., 1990) and have since been found to be widely present in bacteria, where they can be significantly upregulated in response to various adverse environmental factors, including temperature stress, nutrient deficiency, oxidative imbalance, ion toxicity, and heavy metal exposure (Kvint et al., 2003). A notable characteristic of USP proteins is the presence of one or more USP domains (Foret et al., 2011). Based on their genetic diversity and sequence homology, USPs in *E. coli* can be classified into four categories, which interact synergistically or antagonistically to perform specific functions in mitigating oxidative stress, iron scavenging, cell motility, and adhesion (Kvint et al., 2003; Nachin et al., 2005).

USPs are also present in plants, having been first identified in rice (Sauter et al., 2002). The number of USP proteins in higher plants varies from several dozen to several hundred, exhibiting remarkable structural and functional diversity. In addition to the typical USP domain, plant USP proteins incorporate various structural domains such as kinase, U-box, zinc finger, Homeodomain leucine zipper (HDzip), and cation exchanger (Chi et al., 2019). This rich array of structural features endows plant USPs with highly diversified functional motifs, enabling them to respond to and resist increasingly complex environmental stresses, including extreme temperatures, drought, hypoxia, and salinity, making them crucial components of the plant adaptability system. For instance, the *Arabidopsis thaliana* AtSUP can respond to heat or cold stress, serving as a molecular chaperone to maintain the activity of target proteins or RNAs, thereby enhancing the stress tolerance of *A. thaliana* (Gonzali et al., 2015; Jung et al., 2015; Kang et al., 2013; Melencion et al., 2017). In tobacco, USPs can recognize phosphorylation signals induced by salinity and osmotic pressure, leading to the activation of target gene expression and the accumulation of osmotic regulatory substances, as well as the maintenance of reactive oxygen species (ROS) homeostasis (Udawat et al., 2016). In *Gossypium arboreum*, the expression of two USPs is induced by drought stress, and they play a role in regulating cellular water content (Maqbool et al., 2008, Maqbool et al., 2009). Beyond their well-established roles in resisting abiotic stresses, USPs also participate in plant responses to biotic stresses. Following infection by pathogens or pathogenic factors, the expression levels of certain USP genes significantly increase or undergo phosphorylation, thereby activating plant defense-related signaling pathways (Chou et al., 2007; Lenman et al., 2008; Merkouropoulos et al., 2008).

Tomato is one of the most significant vegetables globally, and both tomatoes and their processed derivatives are indispensable components of the human diet. Enhancing tomato adaptability holds considerable theoretical and practical value. Recent studies have demonstrated that universal stress proteins (USPs) in tomatoes respond to salt and drought stress, with their overexpression modulating stomatal opening, increasing abscisic acid and chlorophyll content to prevent leaf damage, indicating that USPs contribute to the stress tolerance of tomatoes (Loukehaich et al., 2012; Ijaz et al., 2017).

In contrast to cultivated tomatoes, wild tomatoes typically exhibit higher genetic diversity and enhanced resistance to biotic and abiotic stresses. For instance, wild tomato species such as *Solanum penellii*, *Solanum galapagense*, and *Solanum habrochaites* possess robust salinity, cold, and frost tolerance. Wild tomatoes serve as valuable donors for improving the stress tolerance of cultivated tomatoes and have been repeatedly utilized in breeding programs aimed at enhancing the adaptability of cultivated varieties (Kapazoglou et al., 2023). The assembly and publication of the *Solanum* pangenome have facilitated the integration and utilization of high-adaptability genetic resources from wild tomatoes (Li et al., 2023). Considering the pivotal role of USP genes in plant stress responses, an exploration of the genomic characteristics of the USP gene family within the adaptively diverse *Solanum* genus is of significant value for elucidating the origins of adaptability in wild tomatoes. This study adopts a global perspective at the genus level, identifying 438 USP genes from 13 *Solanum* species. Through phylogenetic analysis and homology inference, we explored the evolutionary trajectory of the USP gene family within *Solanum* and screened four groups of unique USP genes. The USP motifs in *Solanum* are highly conserved, while the number of *cis*-regulatory elements (CREs) in their promoter regions varies. Furthermore, using the pangenome, we identified USP gene groups influenced by structural variants (SVs) and domestication selection. Expression profile data reveals that intronic SVs broadly affect USP expression patterns, leading to alterations in their response capabilities to salt stress. Overall, these findings provide insights into the *Solanum* USP gene family and pave the way for the utilization of high-resistance genetic resources from wild tomatoes.

## Materials and methods

### USP gene family identification

The whole-genome sequences of 13 *Solanum* species were collected from published data (**Supplementary Table S1**). Each species was independently analyzed for the USP gene family. The hidden Markov model (HMM) for the USP domain was downloaded from the InterPro database (http://pfam-legacy.xfam.org/, available on November 10, 2024) with accession number PF00582. We searched for potential USP domains in the whole-genome protein sequences using HMMER (Potter et al., 2018; http://hmmer.org/), considering domain scores with an e-value less than 1e-5 as potential USP genes. Additionally, the USP sequences from *Arabidopsis thaliana* were used as a query in a blastp search against the whole-genome protein sequences, classifying proteins with a similarity rate greater than 30% as potential USP genes. All candidate genes were manually submitted to SMART (https://smart.embl.de/smart/ , available on November 10, 2024) and the CDD database (https://www.ncbi.nlm.nih.gov/Structure/cdd/, available on November 10, 2024) for domain and integrity verification. Candidates lacking the USP domain, having incomplete USP domains, or with an amino acid length of less than 100 were removed, resulting in the final list of potential USP genes in *Solanum*.

### Phylogenetic tree construction

The phylogenetic tree was constructed using all USP amino acid sequences. Multiple sequence alignment (MSA) was performed with Muscle using default parameters (Edgar, 2004). The MSA results were trimmed using trimAL (Capella-Gutiérrez et al., 2009), removing columns with a gap rate greater than 20% and a consistency ratio lower than 0.001. The trimmed MSA was then used to build the phylogenetic tree with IQ-TREE (version 2) employing the maximum likelihood method (Minh et al., 2020). The optimal model was automatically calculated by the software, with 1000 bootstrap replicates. Tree visualization and enhancement were performed using iTOL (https://itol.embl.de/, available on November 11, 2024).

### Homology and synteny analysis

We conducted synteny analysis using McscanX (Wang et al., 2012), comparing all species pairwise to identify syntenic regions with an e-value of 1e-5 and a minimum of 10 blast hits. The output results were manually organized based on the order of species divergence, and synteny visualization was performed using TBtools (version 2) (Chen et al., 2023). Homology screening and orthogroup classification of all USP genes in *Solanum* were conducted using OrthoFinder2 (Emms and Kelly, 2019; https://github.com/davidemms/OrthoFinder). The coding sequences (CDS) of all direct homologous genes in each orthogroup were analyzed for *Ka* and *Ks* values using KaKs_Calculator (version 3) (Zhang, 2022). The distribution of *Ka/Ks* values was visualized using the R package ggridges (https://github.com/wilkelab/ggridges).

### Motif and *cis*-regulatory element screening

The amino acid sequences of USP proteins from wild and cultivated tomatoes were analyzed for motifs using MEME Suite (v4.12.0) (Bailey et al., 2006), with a minimum motif length of 6, a maximum length of 50, a maximum of 10 motifs, and an e-value threshold of 1e-5. A 2 kb upstream region of all *Solanum* USP genes was extracted as the promoter region, and the USP promoters for both wild and cultivated tomatoes were submitted to the PlantCARE database (https://bioinformatics.psb.ugent.be/webtools/plantcare/html/, available on November 11, 2024) for *cis*-regulatory element (CRE) prediction.

### Graph pan-genome construction and structural variation visualizations

Utilizing the PGGB pipeline (Garrison et al., 2024, https://github.com/pangenome/pggb), each tomato variety was treated as a haplotype, and the graph pan-genome was constructed by chromosome. The percent identity for mapping/alignment was set to 90%, with a minimum block length filter for mapping of 90 bp and a k-mer size for mapping of 19, while all other parameters were maintained at their default settings. The cultivated tomato (SL5) served as the reference path within the graph pan-genome. Structural variants (SVs) were deconstructed using VG (Garrison et al., 2018, https://github.com/vgteam/vg), with a maximum variant length of 100 kb, deconstructing both top-level snarls and nested snarls. The ODGI tool (Guarracino et al., 2022, https://github.com/pangenome/odgi) was employed to extract the graph structure of the USP gene body region and to generate a 2D visualization. Subsequently, SVs within the filtered USP gene body were displayed using Bandage (Wick et al., 2015).

### The domestication selection of USP genes

Using previously published data from natural tomato populations, analyses of domestication selection and genetic diversity were conducted (National Center for Biotechnology Information accession: SRP045767). BWA-MEM was utilized to align resequencing data from cultivated and wild tomato populations against the cultivated tomato reference genome (SL5.0) (Li, 2013). Samtools was employed for BAM file sorting and index creation (Li et al., 2009). SNP calling was performed using DeepVariant (https://github.com/google/deepvariant) in WGS mode on the sorted BAM files (Poplin et al., 2018). VCF merging was accomplished using Glnexus, configured to DeepVariant_unfiltered (Yun et al., 2021). Multi-allelic variants, with a minor allele frequency (MAF) of less than 0.05, a missing rate greater than 0.2, or a depth exceeding 1.8x or falling below 0.3x of the average depth, were filtered out. Based on previously published results regarding domestication and improvement segments in cultivated tomatoes (Lin et al., 2014), and the positional information of USP genes, USP genes subjected to domestication selection were identified. Candidate domestication selection genes were evaluated for pi values using Vcftools (Danecek et al., 2011), and R was utilized to create a line graph visualizing the pi values for cultivated and wild tomatoes.

### Tissue-specific and salt stress expression analysis

Expression analysis was conducted for cultivated tomato (*Solanum lycopersicum*) utilizing publicly available, standardized transcriptome databases. USP tissue-specific expression data were collected from the Tomato Functional Genomics Database (TFGD: http://ted.bti.cornell.edu/cgi-bin/TFGD/digital/home.cgi, available on November 10, 2024). Additionally, expression levels of USP in the roots of cultivated and wild tomatoes under salt stress were obtained from previously published transcriptomic data (CHINA CITIC BANK CORPORATION LIMITED accession: CRA004289) (Li et al., 2022). All expression levels were initially standardized, followed by visualization through a heatmap, which was generated using the R package complexHeatmap (Gu, 2022).

## Result

### The identification and classification of USP gene in *Solanum*

**Figure 1:**
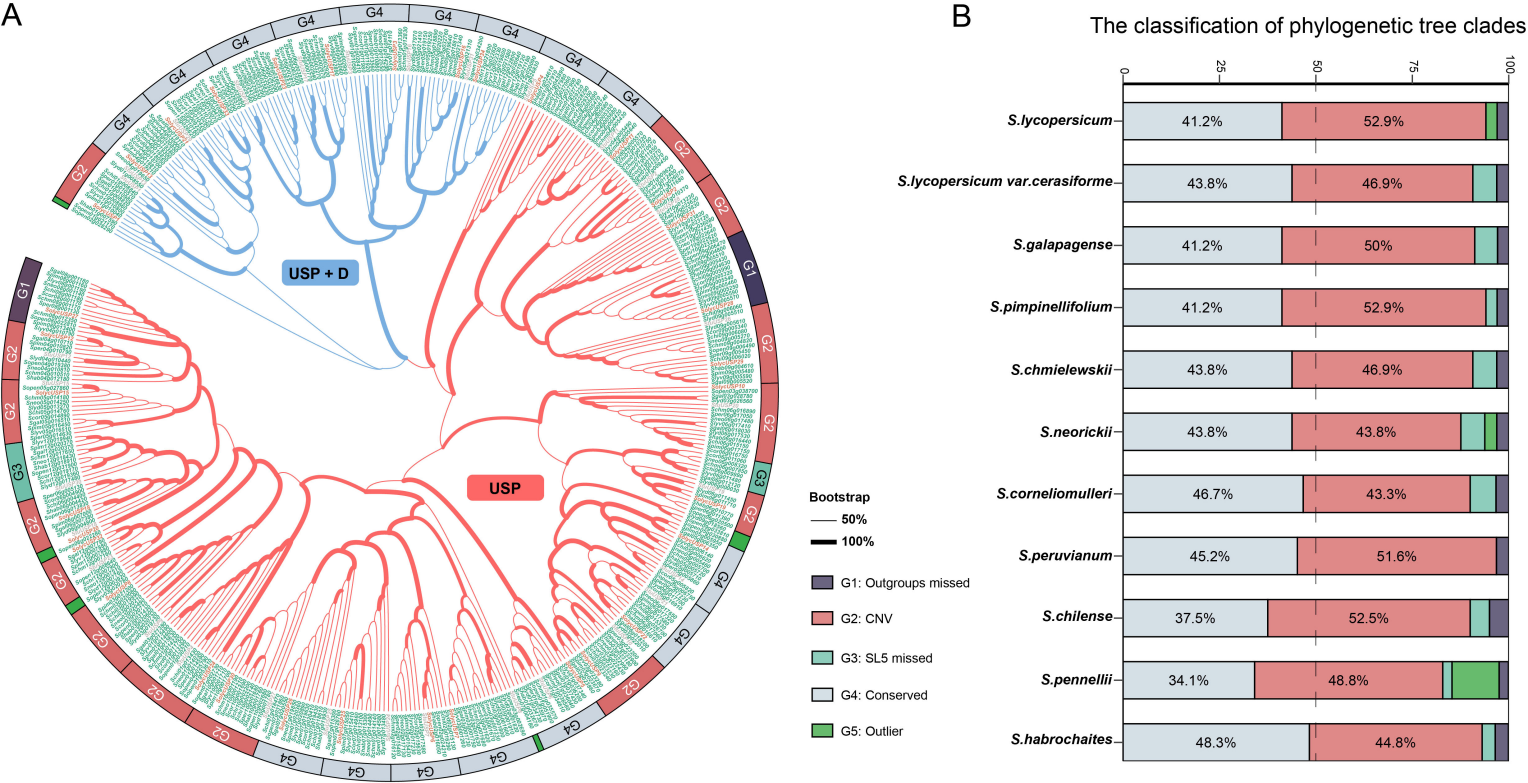
The phylogenetic tree and classification of USP protein. **(A)** USP phylogenetic tree. The clade thickness of the evolutionary tree represents the bootstrap level. The clades of different subfamilies are distinguished by different colors. The USP label of the outgroup is labeled in gray, the USP label of SL5 (*S.lycopersicum*) is labeled in red, and the USP labels of the remaining tomatoes are labeled in green. Different subgroups from G1-G5 were labeled with different colored rectangles at the periphery of the evolutionary tree. **(B)** Statistics of the percentage of USPs of different subgroups in each species.

Domain identification and homology comparison methods were simultaneously employed for the identification of USP genes. Following the confirmation of domain integrity, a total of 438 potential USP genes were collected from *Solanum* (**Supplementary Table S2**), with 31 sourced from potato (considered as outgroups), 66 from *S. lycopersicum* and *S. lycopersicum* var. *cerasiforme* (designated as cultivars), and 300 from eight wild tomato and related species (designated as wild). Among the cultivated tomatoes, 34 potential USP genes were identified, which were renamed as *SolycUSP1* to *SolycUSP34* based on their chromosomal positions and order. All these USPs were assigned to a likelihood tree (**Figure 1A**), where bootstrap analysis supported the robustness of the major clades and branches. Phylogenetic analysis indicates that the USP genes in *Solanum* are primarily divided into two categories based on their domain characteristics: 333 USPs (approximately 76% of all USPs) possess a single typical USP domain, occupying the predominant portion of the evolutionary tree (designated as the USP subfamily), while 105 USPs (approximately 24% of all USPs) occupy another clade, possessing one or more additional domain types alongside the USP domain, primarily protein kinases (designated as the USP+D subfamily).

The phylogenetic tree was divided into 39 groups (**Figure 1A**), with each branch representing a unit based on the respective *SolycUSP*, specifically: Group G1 lacks USPs from the outgroup species (potato); USPs in Group G2 exhibit CNVs; Group G3 is devoid of USPs from S. *lycopersicum*; USPs in Group G4 are highly conserved; and Group G5 comprises outlier USPs. These groupings generally correlate with phylogenetic lineages but also exhibit nesting within different clades. USP27 and USP28 represent two unique groups of USP genes in tomato, with outgroup missing in this group (G1). In the two G3 groups, the absence of cultivated tomato USPs is noted; one G3 group comprises solely wild tomatoes, while the other includes both wild tomatoes and outgroup, with *USP18* and *USP19* being the closest relatives in the phylogenetic tree, suggesting their potential loss during the evolution of cultivated tomatoes. The five G5 group USPs are distributed across isolated branches, with USPs assigned from cultivated tomatoes, wild tomatoes, and outgroup. Sixteen G4 groups (approximately 41% of the total number of groups) exhibit the most conserved phylogenetic characteristics, with each species containing only one USP within these groups. Fifteen G2 groups (approximately 38% of the total groups) show varying degrees of interspecific gene copy number variation (CNV), with some wild tomato USPs exhibiting increases or decreases in gene numbers. G2 may represent USPs in tomatoes that have been subjected to evolutionary perturbation. Based on this grouping, the types and proportions of USPs in each tomato species were statistically analyzed (**Figure 1B**). The G2 and G4 groups comprise the majority of all tomato USPs, where, except for S. neorickii, *S. cornelimulleri*, and *S. habrochaites*, G2 group USPs exceed G4 group USPs in most tomatoes, indicating widespread CNV of USPs in tomatoes. Among the remaining three groups, G5 is distinct, primarily concentrated in the USPs of *S. pennellii*, suggesting a unique differentiation of USPs in this species.

### The collinearity analysis of USP gene in *Solanum*

**Figure 2:**
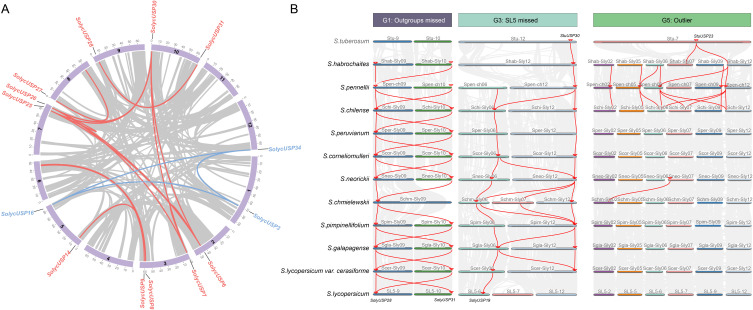
Colinearity analysis of USP gene. **(A)** Cultivated tomato species USP covariance analysis. USPs of the USP subfamily are labeled in red and USPs of the USP+D subfamily are labeled in blue. Red connecting lines represent the presence of covariance. **(B)** Analysis of USP covariance among multiple species of the genus Tomato. Species order is in the order of their divergence. Red linkage represents the presence of covariance. USP genes are identified by small red arrows.

Collinearity analysis reveals the presence of four pairs of collinear gene pairs in cultivated tomatoes (**Figure 2A**), among which the USP subfamily includes a pair of collinear genes *USP3/16/34*, while the USP+D subfamily comprises three pairs of collinear genes: *USP6/7/25/26/30*, *USP9/14*, and *USP28/31*. All collinear genes are generally located at different positions on different chromosomes, indicating that segmental duplication predominantly drives the expansion of *SolycUSPs*. Furthermore, the collinearity changes of USPs among tomatoes were explored based on the divergence order of all species. The USPs in G2 and G4 groups exhibited good collinearity across different tomatoes, particularly G4 group USPs, which demonstrated nearly complete conservation of collinearity among species, highlighting the highly conserved nature of USPs in G4 (**Supplementary Figure S1**). We focused on the G1, G3, and G5 groups, where collinearity exhibited variation among species (**Figure 2B**). The G1 group includes USP28 and USP31; with the exception of *S. chmieliewskii*, these two USPs maintain stable collinear relationships in other tomatoes. In cultivated tomatoes, they form a pair of collinear gene pairs, while potato USP is absent from this group. *SolycUSP28*, located on a branch in the phylogenetic tree, lacks potato USP, whereas *SolycUSP31* shares the same branch as StuUSP, suggesting that after the divergence of potato and tomato ancestors, SolycUSP31 underwent segmental duplication to produce the tomato-specific *SolycUSP28*, which subsequently lost collinearity with potato during further divergence. The G3 group contains *StuUSP30*, which underwent duplication on chromosome 6 after the divergence of *S. habrochaites* and *S. pennellii*, resulting in *USP19*. Both genes were retained in wild tomatoes, while the collinear *SolycUSP* associated with the original *StuUSP30* was lost in cultivated tomatoes, corresponding to the G4 group in the phylogenetic tree, where *USP19*, derived from duplication, remains adjacent to the G4 group branch. G5 group USPs primarily emerged and expanded in *S. habrochaites*, *S. pennellii*, and *S. chilense* tomatoes formed during early divergence, subsequently disappearing in later tomatoes. Although these USPs retained collinear relationships in the early three tomatoes, they were only identified as candidate USPs in *S. pennellii*.

### The Ka/Ks analysis of orthologues USP gene in *Solanum*

**Figure 3:**
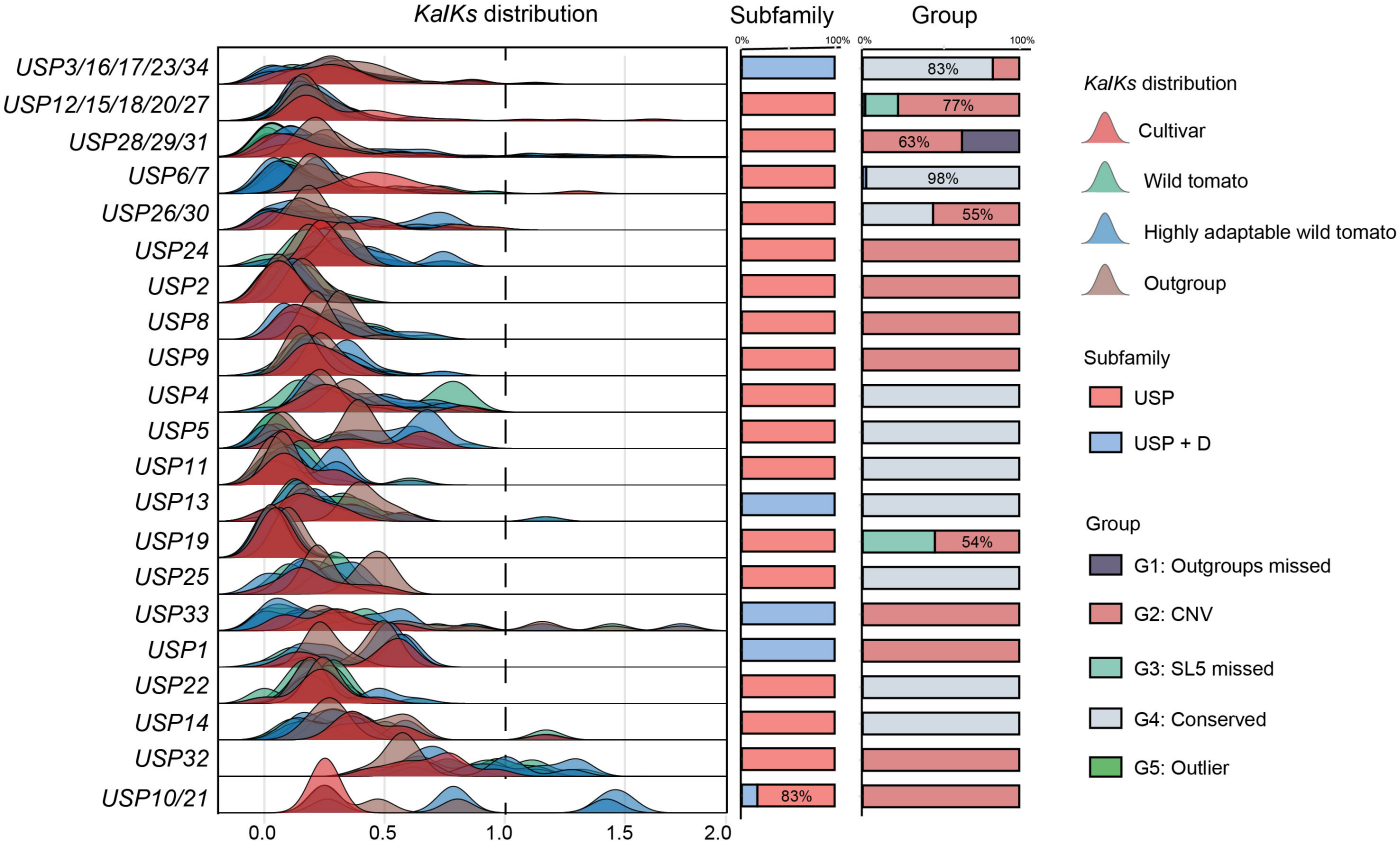
The distribution of Ka/Ks values of USP orthogroup. orthogroups are represented according to the USP proteins they contain. The distribution of different groups of tomatoes is distinguished by different colors.

To clarify the extent of evolutionary constraints on USPs in *Solanum*, we identified the direct and paralogous relationships among all potential USP genes, categorizing them into 21 orthogroups. We calculated the *Ka* and *Ks* values between all orthologues within each orthogroup (**Figure 3**). Each orthogroup was designated based on the USP genes it contained from cultivated tomatoes. Notably, several tomato species with broad or strong resistance to environmental stress (*S. pennellii*: tolerant to salt and drought stress; *S. habrochaites*: tolerant to cold stress; *S. galapagense* and *S. chmielewskii*: tolerant to salt stress) were classified as highly adaptable wild tomatoes. The results reveals that in 17 orthogroups, the *Ka/Ks* ratio for USPs across all species was significantly less than 1, indicating that the majority of USP genes are subjected to stringent negative selection, leading to a stabilization of gene function throughout evolution. In orthogroups containing *USP33*, *USP32*, and *USP10*/*21*, some genes exhibited *Ka*/*Ks* values greater than 1, indicating they are under positive selection. These genes belong to the G2 group and are primarily derived from highly adaptable wild tomatoes, suggesting that CNV may have driven the evolution of USPs to some extent, with multi-copy redundancy facilitating gene neofunctionalization or sub functionalization, thus potentially forming the genomic basis for the high adaptability of wild tomatoes.

### The motif and CREs analysis of USP gene in tomato

**Figure 4:**
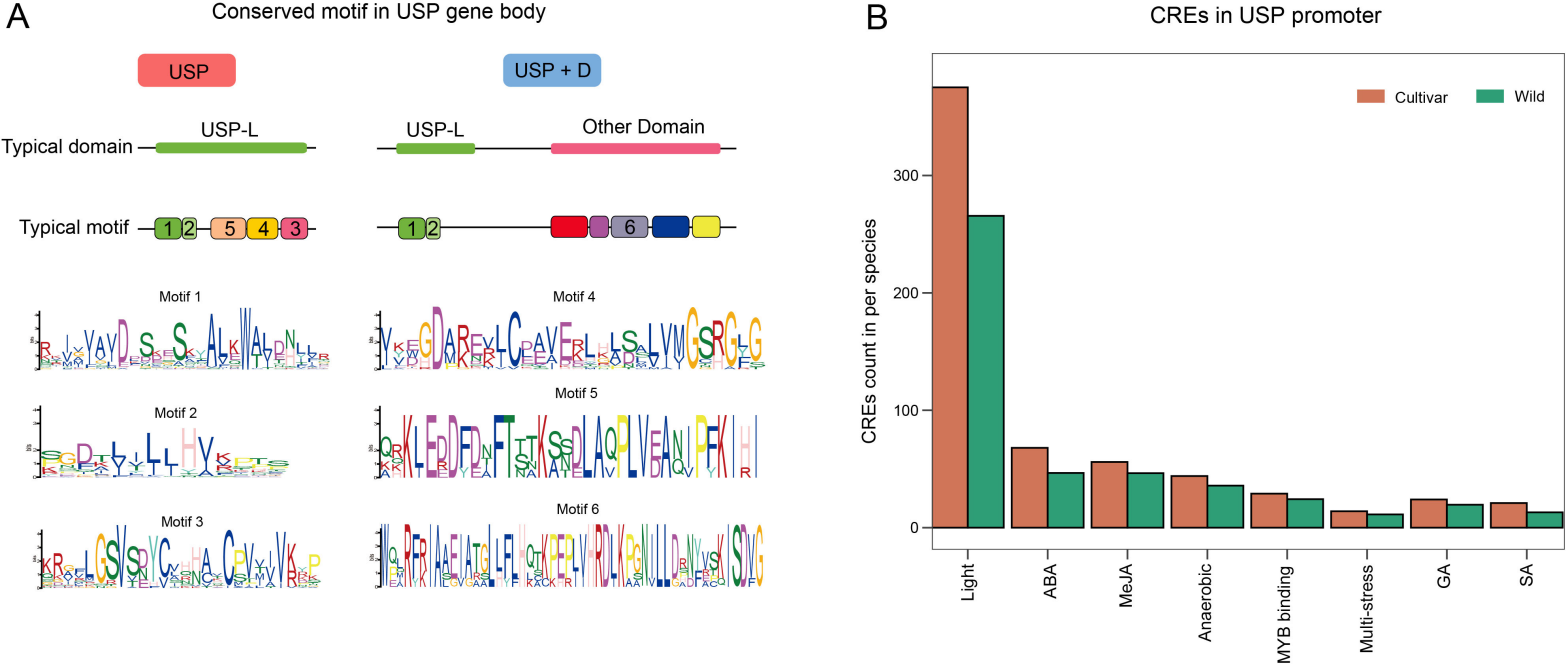
The motif and promoter CREs analysis of USP gene. **(A)** USP gene Typical structural domains and motifs. different motifs are indicates by different colors and numbers, motifs without numbers are motifs that are not conserved in wild and cultivated tomato7-10. motif1–6 concordant sequences are shown, x-axis indicates the sequence order, and the y-axis and the size of the amino acid characters indicate the degree of conservation of the sequence in the motif. **(B)** Classification and number statistics of promoter CREs in cultivated and wild tomato. x-axis is the type of CREs and y-axis is the average number of CREs in each species.

Motifs are closely related to protein function. We independently searched for and predicted 10 motifs from all USPs in both wild and cultivated tomatoes (**Figure 4A**). The independent prediction results indicates that six motifs are highly conserved between wild and cultivated tomatoes, while four motifs exhibit considerable variability. The highly conserved motifs 1–5 form the USP domain, whereas the conserved motif 6 and the remaining four variable motifs are concentrated in the variable domains of the USP+D subfamily. This suggests that there may not be a significant functional differentiation of USP genes between wild and cultivated tomatoes, given their stable and consistent motif distribution. The functional differences of USP genes in tomatoes appear to be primarily concentrated in the USP+D subfamily, where the USP proteins possess diverse structures to cope with complex stress environments, potentially leading to substantial variation among different tomato species. Notably, although both subfamilies contain a USP domain, motif analysis reveals that the USP domain characteristics seem to differ; USPs in the USP+D subfamily possess only motifs 1 and 2, lacking motifs 3, 4, and 5.

CREs play a crucial role in triggering TFs to bind and regulate gene expression. We independently searched for and predicted CREs in the promoters of all USP genes from wild and cultivated tomatoes (**Figure 4B**). Among the top ten ranked CREs, the types of CREs were completely consistent between wild and cultivated tomatoes. Except for those associated with light and MYB binding, all other CREs were related to hormone or environmental responses, aligning with the functional role of USPs in responding to various stress conditions. There are significant differences in the number of CREs among different tomato species, with cultivated tomato USP promoters exhibiting a greater average number of all CRE types compared to wild tomatoes. This indicates that while USPs in different tomato species may respond to similar environmental factors, their expression levels and patterns differ significantly.

### The SVs and domestication of USP gene in tomato

**Figure 5:**
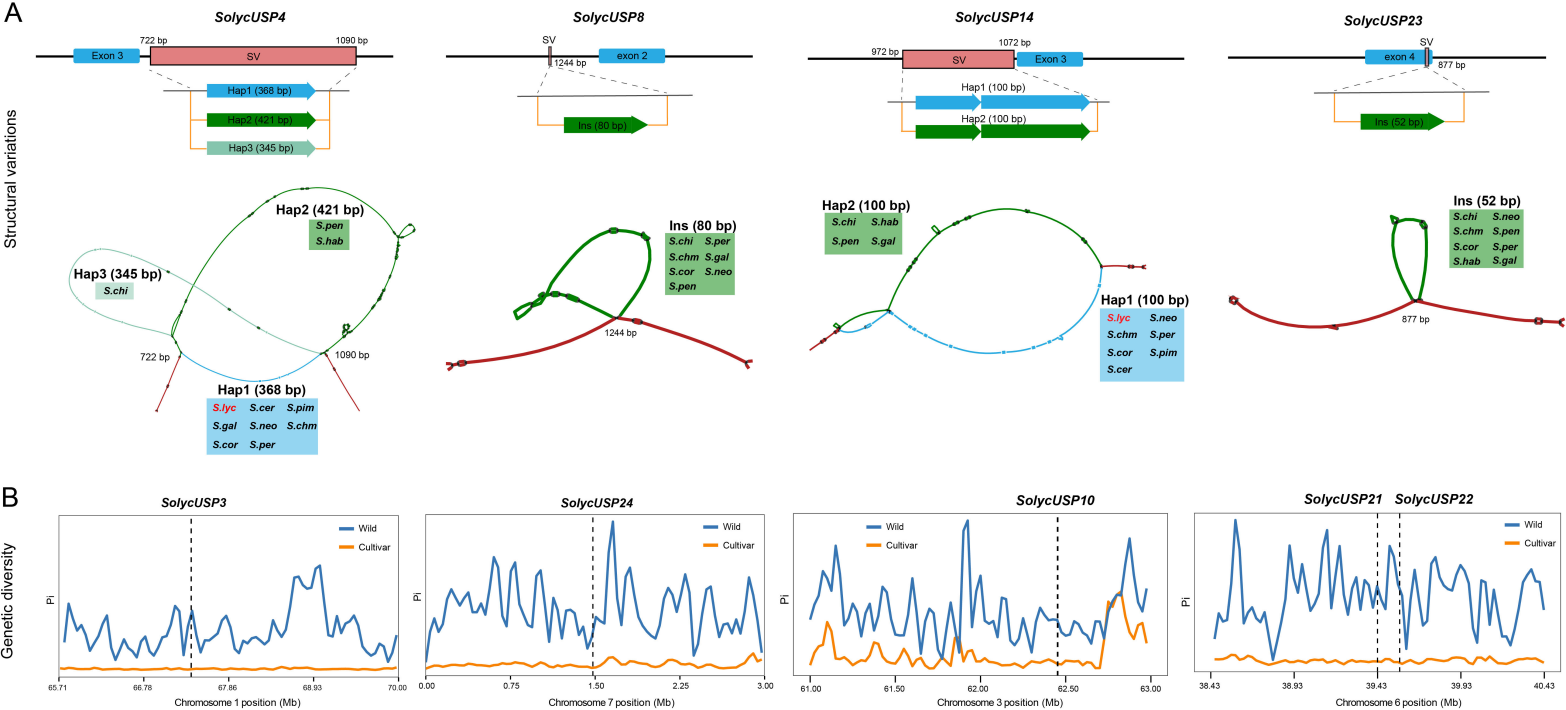
The SVs and domestication of USP gene. **(A)** Schematic diagram of USP gene body SVs locations and types. Above is a structural map of the part of the genome containing SVs. The start and end positions of the structural variants are relative to the position of the initiation start codon (1-base). Below is a visualization of the graph structure corresponding to SVs, with red representing the concordance path, different variant paths are indicates by different colors, and tomatoes supporting the variants are labeled at the corresponding positions. **(B)** The distribution of pi values of domesticated and improved USP genes. x-axis is the genomic position, y-axis is the corresponding pi value of the gene, orange is the pi value of cultivated tomato, blue is the pi value of wild tomato, and the grey dotted line marks the position of the gene.

To characterize the variation in the USP gene regions, we followed the PGGB pipeline to generate a graph pangenome that includes the whole genomes of two cultivated tomatoes and nine wild tomatoes (**Supplementary Table S3**), thereby deconstructing the variations among all tomato species. Due to the high genetic diversity of wild tomatoes, all USPs were found to be covered by variations. SVs typically have greater causal effects, so we focused on SVs supported by multiple tomato species within the USP gene body regions, avoiding overly complex SVs that may arise from graph construction or other factors. Ultimately, 11 USP genes were identified as having stable and reliable SVs between wild and cultivated tomatoes (**Supplementary Table S2**), with these variations primarily located in the intronic regions of USPs. We illustrate the SVs for four of these genes (**Figure 5A**): in the *SolycUSP4* region, a 368 bp polymorphic interval exists between the third and fourth exons, supported by *S. pennellii* and *S. habrochaites* for Hap2, and by *S. chilense* for a unique Hap3. In *SolycUSP14*, a 100 bp polymorphic interval is present upstream of the C-terminus of the third exon, with *S. chilense*, *S. habrochaites*, *S. pennellii*, and *S. galapagense* supporting a different Hap2 compared to other tomatoes. Additionally, within the gene regions of *SolycUSP8* and *SolycUSP23*, there are insertions of 80 bp (supported by seven wild tomato species) and 52 bp (supported by eight wild tomato species), respectively, with the insertion in *SolycUSP23* occurring within its fourth exon.

Previous studies have identified potential genomic segments in cultivated tomatoes that are influenced by domestication and improvement. Among all USPs, *SolycUSP3* and *SolycUSP24* are located within segments under domestication selection, while *SolycUSP10*/*21/22/28/29/30* are situated within segments under improvement selection. To verify the selection pressures on USPs, we generated Pi value distributions for cultivated and wild tomatoes from natural populations to observe changes in genetic diversity (**Figure 5B**). Compared to wild tomatoes, the eight USPs subjected to domestication or improvement exhibited a dramatic reduction in genetic diversity in cultivated tomatoes, indicating that these USPs underwent strong selection during the domestication process.

### The expression analysis of USP gene in tomato

**Figure 6:**
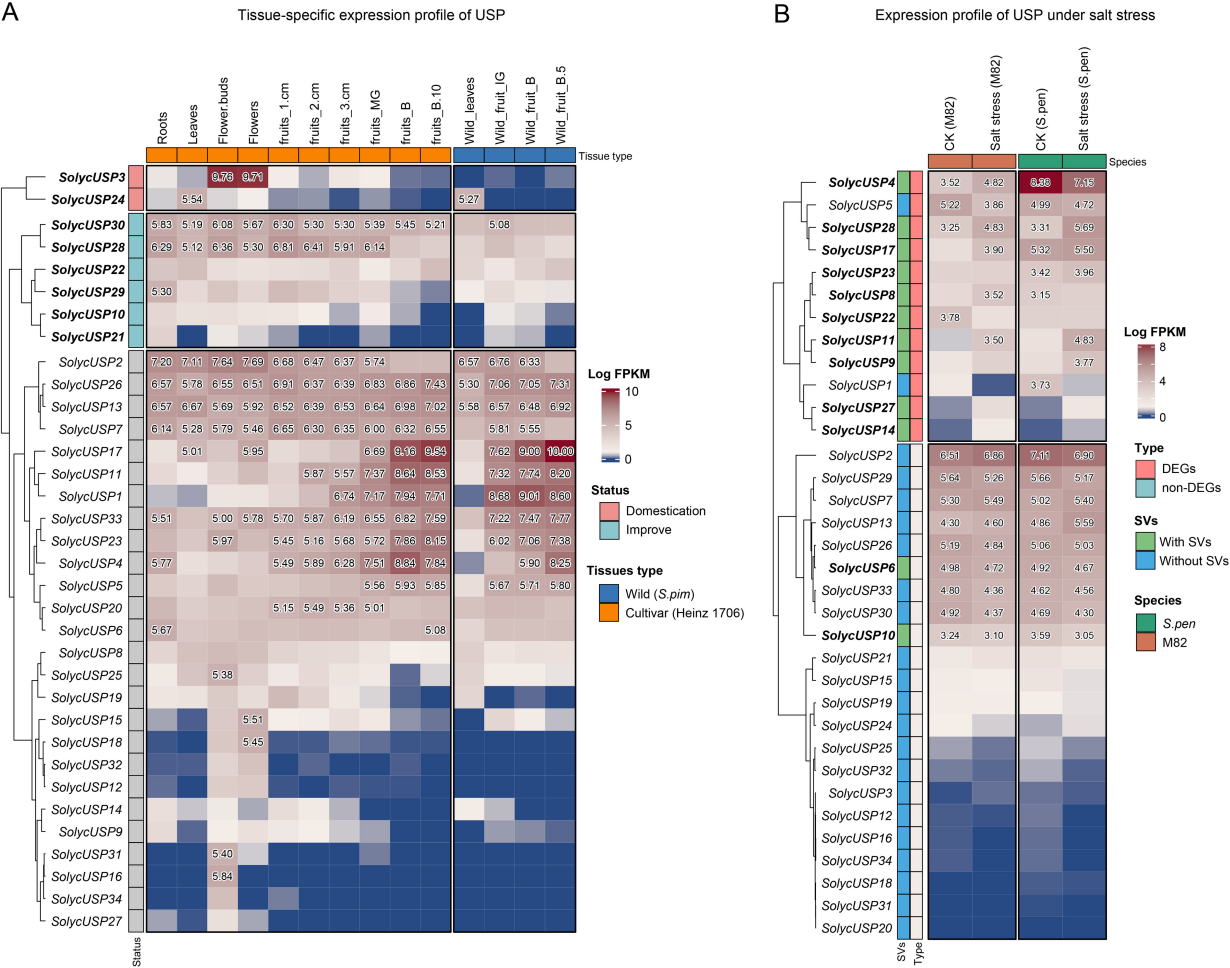
The tissue-specific and salt-response expression profile of USP gene. **(A)** Heatmap of tissue-specific expression of USP. Expression was processed using log2 and numbers greater than 5 are displayed. The status of genes subjected to domestication selection is indicates at gene id. **(B)** Heatmap of USP expression in response to salt stress. Expression was processed using log2 and numbers greater than 3 are shown. Whether genes were shown significant expression differences and whether they had SV is shown at gene id.

Using publicly available expression data, we generated a tissue-specific expression heatmap for *SolycUSPs* (**Figure 6A**). Among the two genes affected by domestication selection, *SolycUSP3* was expressed in vegetative organs and early fruit development, with particularly high specificity in flowers and buds. *SolycUSP24* exhibited specific expression mainly in vegetative organs, especially in leaves. Domestication selection has led to a reduction in the expression levels of both *SolycUSP3* and *SolycUSP24*. This effect is particularly pronounced among the six genes subjected to improvement selection, with *SolycUSP30* and *SolycUSP28* highly expressed in nearly all cultivated tomato tissues, while only *SolycUSP20* showed some expression in the leaves of wild tomatoes. *SolycUSP10* and *SolycUSP21*, located in positively selected orthogroups, are also considered affected by improvement selection. Notably, while these two genes exhibit low expression in the fruit of wild tomatoes, their expression increases in cultivated tomatoes. Among the USPs not subjected to domestication or improvement selection, three primary expression patterns were observed: four *SolycUSPs* (*SolycUSP2/26/13/7*) displayed high expression across nearly all vegetative and reproductive organs; six SolycUSPs (*SolycUSP1/4/11/17/23/33*) were highly expressed primarily in fruits, particularly in fully mature fruits, with similar expression patterns in both cultivated and wild tomatoes. Additionally, ten SolycUSPs were expressed almost exclusively in flowers or flower buds (*SolycUSP15 to SolycUSP27*), showing little to no expression in other tissues. Furthermore, structural variations did not significantly impact the tissue-specific expression of USPs.

### SV probably enhanced tomato USP response to salt stress

We explored the response of USPs to salt stress using previously published RNA-seq data from the roots of cultivated tomato (M82) and wild tomato (*S. pennellii*) under salt stress (**Figure 6B**). More than half of the *SolycUSPs* were highly expressed in response to salt stress, with 12 *SolycUSPs* showing significant differences in expression levels between cultivated and wild tomatoes. Under salt stress conditions, nearly all differentially expressed genes (DEGs) exhibited increased expression in wild tomatoes compared to cultivated tomatoes, indicating that the high salt tolerance of *S. pennellii* may be associated with the increased mRNA levels and protein abundances of *SpenUSPs*. Notably, among the 11 USPs identified as having stable and reliable structural variations, 9 exhibited differential expression between cultivated and wild tomatoes; only *SolycUSP5* and *SolycUSP1* did not contain SVs. This may suggest that haplotype variations, deletions, and insertions of USPs significantly influence their expression responses to stress. Nine *SolycUSPs* (*SolycUSP2* to *SolycUSP10*) were highly expressed but did not respond to salt stress, showing no expression differences between cultivated and wild tomatoes, These 9 high expressed genes exhibited constitutive expression in the roots of tomatoes. Furthermore, 13 *SolycUSPs* showed minimal or low expression in both cultivated and wild tomatoes, and they were also not induced by salt stress.

## Discussion

It is generally accepted that modern tomatoes encompass 16 wild species or closely related wild relatives. Extended evolution, domestication, and breeding efforts have significantly enriched the phenotypic and genotypic diversity of tomatoes (Li et al., 2018; Peralta and Spooner, 2024). The assembly and publication of the pan-genome of the *Solanum* have greatly accelerated the process of utilizing wild tomato resources for the enhancement of modern tomato breeding, facilitating genomic studies across the entire genus (Li et al., 2023). Our research focuses on the USP gene family, which is closely associated with adaptability—the primary objective of contemporary tomato breeding. Initially identified in bacteria, these genes are characterized by the presence of a single USP domain, whose function is believed to be related to oxidative stress defense and iron scavenging (Nachin et al., 2005). Plants exhibit a vast array of USP types and quantities. We identified over 400 potential USPs from the *Solanum*, with the number of USPs per species ranging from 29 to 40. Notably, a previous study on the potato USP gene family collected 108 USP proteins from the entire genome (Qi et al., 2024), a number considerably exceeding that identified in our study. Although variations in gene family counts may arise from differences in genome annotation, identification methods, and filtering criteria, the substantial discrepancy remains unexplained. Our comparative analysis across species ensures that relevant assessments are conducted within the same dimension, thus we maintain our identified count. Most USP proteins in *Solanum* contain only a single USP domain, representing the primary composition, typical features, and conserved functions of the USP family (Vollmer and Bark, 2018). Another category of USPs includes an additional domain; these USPs may arise from the fusion of USP domains with various structural domains prompted by diverse and severe environmental conditions (Tkaczuk et al., 2013). This undoubtedly enhances the diversity of the USP gene family and equips plants to confront increasingly complex adverse stresses. In *Solanum*, this subset of USPs, beyond the typical domain, also encompasses PKs, Serine/Threonine kinases, AANH, and FDF domain. These additional domains may confer capabilities for interaction with other proteins, phosphorylation, and stabilization and folding of protein conformations.

Through phylogenetic analysis of all USP proteins, we discovered that copy number variation (CNV) is widely present among USPs of *Solanum*. CNV affects gene numbers through mechanisms such as whole-genome duplication, retention, and segmental duplication. It holds significant implications for species evolution, environmental adaptability, specific gene expression, and regulation, and has been extensively studied (Freeman et al., 2006; Zarrei et al., 2015). As observed in other species, the prevalent CNVs among different tomatoes may be closely related to their adaptive differences (Cho et al., 2023; Kumar et al., 2023; Stalder et al., 2023). Notably, the USP group G5 is uniquely concentrated in *S. pennellii*, exhibiting collinearity with some genes in *S. habrochaites* and *S. chilense* that lack the typical characteristics of USP domains, which subsequently disappeared during the evolutionary process of tomatoes. *S. pennellii* is renowned for its robust drought, salt, pest, and disease resistance, making it a primary genetic resource and foundation for adaptive breeding in cultivated tomatoes (Bolger et al., 2014). This suggests an intriguing hypothesis that the exceptional resistance traits of *S. pennellii* may be associated with the specific enrichment of unique USPs within this species.

Graph pangenomes represent the forefront of genomic development and serve as a powerful tool for elucidating SVs, having been widely employed in studies of genetic evolution and association mapping (Groza et al., 2024; Khan et al., 2024; Liu et al., 2024). Constructing high-quality and reliable plant graph pangenomes remains challenging due to the complexity of genomic structures and the slow development of corresponding tools. Rather than exploring the global perspective of graph pangenomes, focusing on specific important genomic regions or gene bodies may be more suitable given the current state of graph pangenome development. Such methodologies have been successfully applied in areas such as genomic structure, locus evolution, adaptive breeding, and evolutionary studies, yielding significant advancements (Bolognini et al., 2024; Jamsandekar et al., 2024; Jayakodi et al., 2024). Our research similarly concentrates on variations within the USP gene body regions. To avoid false-positive variations arising from structural complexities, we excluded USP genes with highly convoluted graph structures, ensuring that SVs are supported by multiple haplotypes. These SVs were confirmed through 2D visualization of graph regions. We identified eight USPs covered by stable SVs, which include haplotype variations, insertions, and deletions, with each SV also containing several minor nested variations. This undoubtedly enhances the genetic diversity and heterogeneity of USPs, offering opportunities for their utilization. Notably, SVs predominantly reside within the intron regions of USPs. Analysis of expression profiles from cultivated and wild tomatoes under salt stress indicates that these SVs significantly influence the expression patterns of the corresponding USP genes. Similar phenomena have been observed in other studies, where SVs may affect alternative splicing processes or alter the status of regulatory elements such as enhancers, thereby significantly impacting mRNA abundance of target genes, particularly in complex quantitative traits (Pagani and Baralle, 2004; Chiang et al., 2017; Beird et al., 2024). We hypothesize that these SVs may arise from the balance between evolution and natural selection, adjusting the SVs within USPs to enhance or diminish gene function, thus better adapting to complex environments while avoiding the pressure to generate new genes. It is essential to note that accurately resolving SVs using graph structures constructed from multiple haplotype genomes remains challenging. The breakpoint positions and chimeric variations of these SVs may not align with true biological realities. The conclusions presented in this study are predominantly descriptive. Therefore, we strongly recommend that subsequent research endeavors aimed at validating these hypotheses incorporate rigorous empirical validation through molecular experimentation or genetic transformation.

## Conclusion

This study systematically identified 438 USP genes across 13 species of the *Solanum*. USP genes among diverse *Solanum* species exhibit widespread CNVs yet are predominantly evolutionarily conserved. The domain architecture and motif composition of *Solanum* USP genes demonstrate considerable consistency, notwithstanding significant variations observed in the abundance of their promoter-associated regulatory elements. Six *SolycUSPs* manifested reduced genetic diversity throughout the domestication process, accompanied by discernible modifications in their tissue-specific expression patterns. Eleven USP genes possess gene bodies affected by high-confidence SVs, which predominantly impact the intronic regions and precipitate widespread alterations in their transcriptional responses to salt stress. *S. pennellii* possesses a greater number of unique USPs, which may be associated with its robust adaptability. Overall, these findings provide valuable insights into the USP gene family within the *Solanum*.

## Data Availability

The original contributions presented in the study are included in the article/**Supplementary Material**. Further inquiries can be directed to the corresponding author/s.

## Supplementary material

Supplementary Figure 1 Colinearity analysis of G2 and G4 group.
