# Supplementary figures and images for: The Evolution and expression analysis of USP gene family in Solanum
Source: Front Plant Sci. 2025 Jun 30;16:1546640. doi: 10.3389/fpls.2025.1546640 (PMC12256548; doi:10.3389/fpls.2025.1546640)

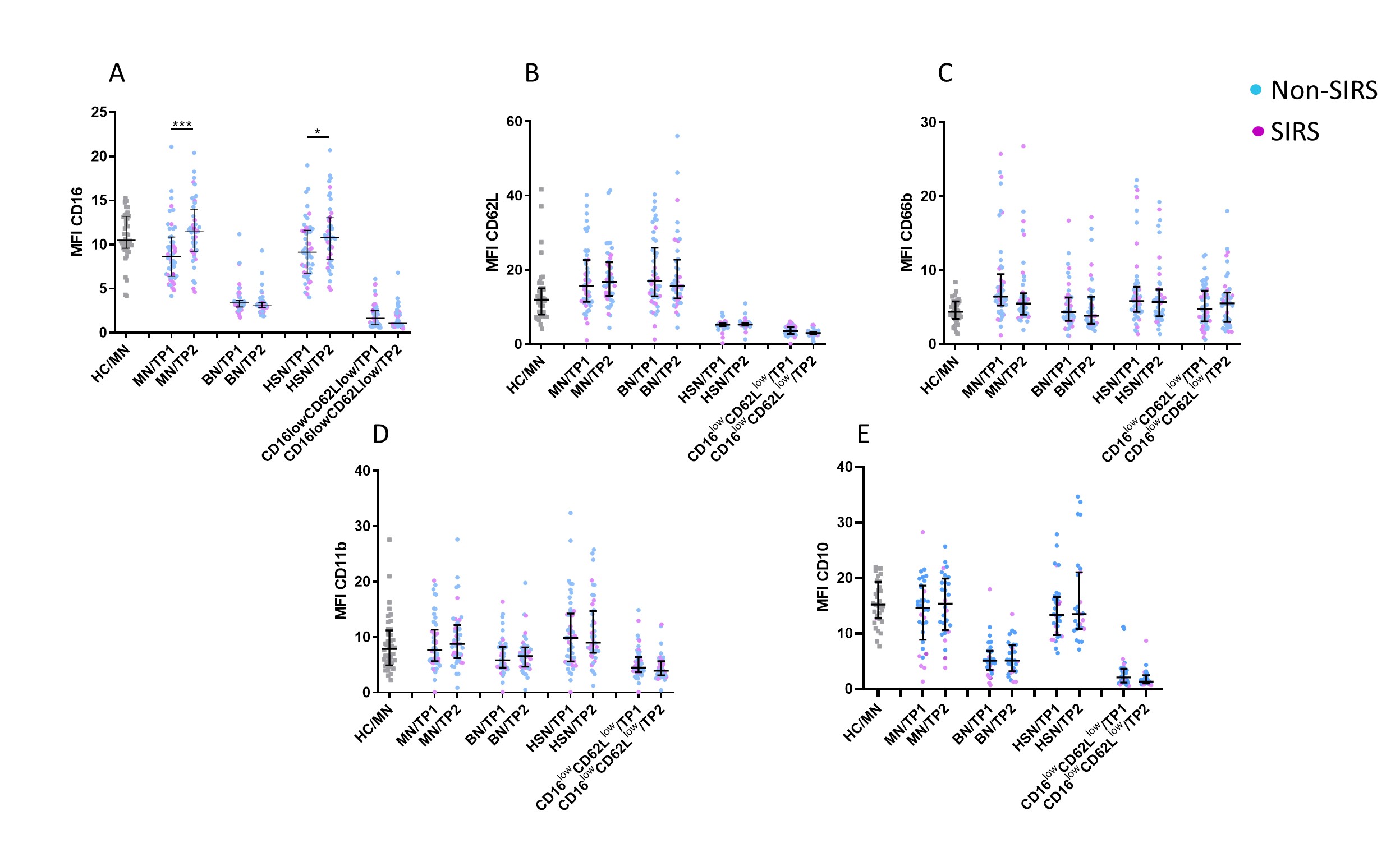

Supplement: Supplementary Figure 1 — Colinearity analysis of G2 and G4 group. [file Image1.jpeg]
